# Evidence for D1 Dopamine Receptor Activation by a Paracrine Signal of Dopamine in Tick Salivary Glands

**DOI:** 10.1371/journal.pone.0016158

**Published:** 2011-01-31

**Authors:** Ladislav Šimo, Juraj Koči, Dušan Žitňan, Yoonseong Park

**Affiliations:** 1 Department of Entomology, Kansas State University, Manhattan, Kansas, United States of America; 2 Institute of Zoology, Slovak Academy of Sciences, Bratislava, Slovakia; New Mexico State University, United States of America

## Abstract

Ticks that feed on vertebrate hosts use their salivary secretion, which contains various bioactive components, to manipulate the host's responses. The mechanisms controlling the tick salivary gland in this dynamic process are not well understood. We identified the tick D1 receptor activated by dopamine, a potent inducer of the salivary secretion of ticks. Temporal and spatial expression patterns examined by immunohistochemistry and reverse transcription polymerase chain reaction suggest that the dopamine produced in the basal cells of salivary gland acini is secreted into the lumen and activates the D1 receptors on the luminal surface of the cells lining the acini. Therefore, we propose a paracrine function of dopamine that is mediated by the D1 receptor in the salivary gland at an early phase of feeding. The molecular and pharmacological characterization of the D1 receptor in this study provides the foundation for understanding the functions of dopamine in the blood-feeding of ticks.

## Introduction

Ticks are obligatory ectoparasites that feed on the blood of vertebrate hosts and often transmit pathogens, including viruses, bacteria, and protozoa. Tick saliva is essential during feeding for the manipulation and suppression of host defense responses and may contain key components in the transmission of pathogens. Biochemical analyses of tick salivary secretions have identified antihemostatic, anti-inflammatory, and immunomodulatory activities [1], . Promising strategies for the prevention of tick blood feeding and for the interruption of pathogen transmission include the disruption of salivary gland (SG) functions.

The SG of female tick is composed of three different types of acini: acini I, II, and III. Each acinus contains several different types of cells, and different categories of secretory vesicles [3], [4]. Acini II and III are considered the major groups that function in producing secretory proteins/compounds and in osmoregulation during feeding, while acinus I is thought to be involved in absorption of water in free-living ticks [5], [6]. For the successful completion of feeding, which requires several days, heterogeneous cells and vesicles in the SG are sequentially activated as follows: 1) secretion of cement to fix and seal the mouth part to the host skin, 2) secretion of antihemostatic, anti-inflammatory, and immunomodulatory salivary components to facilitate the blood feeding and prepare for rapid engorgement toward the end of the feeding phase, and 3) osmoregulation to remove excess fluid during the feeding. Mechanisms underlying the precise control of phase-specific activities of the SG are not well understood.

Control of salivary secretion involves the nervous system. Anatomical and pharmacological studies have implicated several neural components in salivary secretion: dopamine (DA), acetylcholine, and multiple neuropeptides [7], [8], [9]. Among the neurotransmitters/modulators, DA directly stimulates salivary secretion in the isolated SG likely through D1-type receptor [10], while acetylcholine is likely involved in the sensory-mediated processes in the synganglion (brain) that lead to salivary secretion [11]. We previously described peptidergic network in innervation of the SG acini II and III suggesting important roles of neuropeptidergic control of salivary glands. [12],[13].

A model for DA actions on the SG of partially fed ticks has been proposed [14], in which DA activates two independent signaling pathways: cAMP-dependent signal transduction leading to fluid secretions; and a calcium-dependent signaling pathway activating prostaglandin E2 production, which eventually leads to secretion of other protein components in the saliva. Previous studies of DA activity on the SG have mainly focused on the SGs of partially fed ticks using *in vivo* and *in vitro* assays, but this strategy limits the understanding of the roles of DA to only salivary secretion in the specific feeding phase of ticks. In this study, we identified and characterized a DA source and the D1 receptor for DA in the SG of the blacklegged tick *I. scapularis*. In contrast to the presumption of neuronal DA controlling the SG in previous studies (i.e., [7]), our study proposes an alternative or additional function of paracrine DA in the SG during the early feeding phase of ticks and contributes to understanding of mechanisms governing the release of bioactive secretory proteins associated with transmission of pathogens into the host.

## Results

### The gene encoding the dopamine receptor D1

**Figure 1:**
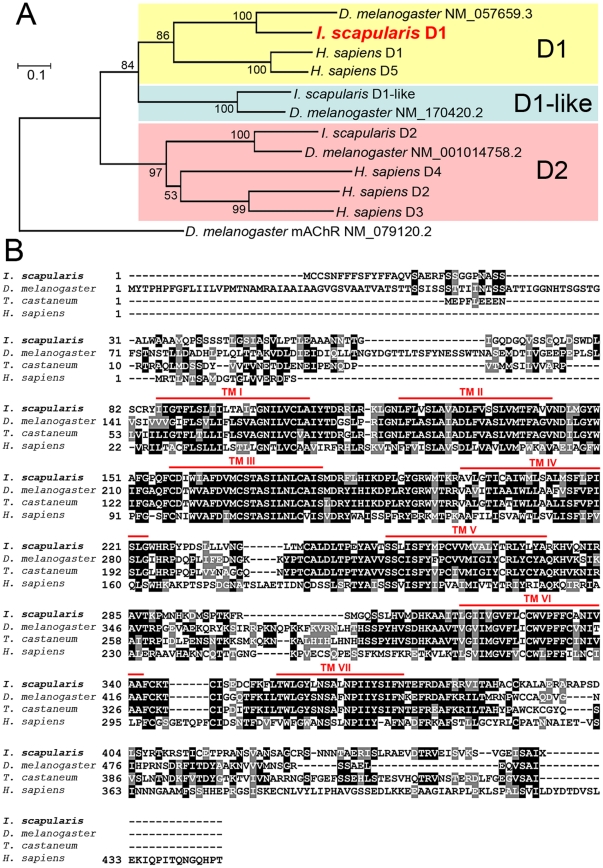
Phylogeny and the amino acid sequences of the tick D1 receptor. (A) Phylogenetic relationship of dopamine receptors including three tick dopamine receptors. The tree was constructed using a neighbor-joining method. Numbers at the nodes are for the percent support using 500 bootstrap replicates. (B) Alignment of conceptual translations for D1 receptor–related sequences. The letters with gray background are similar, letters in the black background are identical amino acids in the sequence alignment using 50% majority rules. Conserved seven-transmembrane segments predicted by TMHMM Server v.2.0 (http://www.cbs.dtu.dk/services/TMHMM/) of *Ixodes* D1 receptor are marked with a solid line (red) above the alignment. GenBank Accession numbers are: *Drosophila melanogaster* D1, NM_057659.3; *D. melanogaster* D1-like, NM_170420.2; *D. melanogaster* D2, NM_001014758.2; *Homo sapiens* D1, NM_000794.3; *H. sapiens* D2, NM_000795.3; *H. sapiens* D3, NM_033663.3; *H. sapiens* D4, NM_000797.3; *H. sapiens* D5, NM_000798.4; *D. melanogaster* muscarinic acetylcholine receptor, NM_079120.2; *Tribolium castaneum* XM_966449; *I. scapularis* D1, XM_002409243.1; *I.scapularis* D1-like, XM_002399612.1; and *I. scapularis* D2, XM_002416405.1. The *I. scapularis* sequence was extended to the 5′ end based on our data.

Phylogenetic analyses comparing the *I. scapularis* D1 receptor with *Drosophila* (D1, D1-like, and D2) and human (D1–D5) dopamine receptor sequences demonstrated a clear orthologous cluster in the *D1 group*, including human D1 and D5 (Figure 1A). The tick D1 receptor is the ortholog of *Drosophila* NM_057659.3, which was previously shown to be involved in the DA-induced elevation of cAMP in a heterologous expression system [15]. Another tick G protein-coupled receptor (GPCR), tentatively named D1-like, is orthologous to *Drosophila* NM_170420.2, in the group closely related to the *D1 group*, but without vertebrate counterparts. The third tick GPCR was clearly categorized into the *D2 group* according to the sequence similarities to *Drosophila* NM_001014758.2 and human D2, D3, and D4 (Figure 1A). The GenBank Accession Numbers of the sequences for this analysis are in the Fig. 1.

We were able to obtain the full-length open reading frame (ORF) encoding the D1 receptor, The primers designed on each putative start (ATG) and stop (TAG) codons amplified the full-length ORF. RNA template was treated with DNase to avoid the contamination by genomic DNA in the reverse transcription-PCR of this intronless gene. The gene encoding *I. scapularis* D1 receptor encodes 461 amino acid residues containing seven putative transmembrane segments (Figure 1B).

### Dopamine production in basal cells of acini

**Figure 2:**
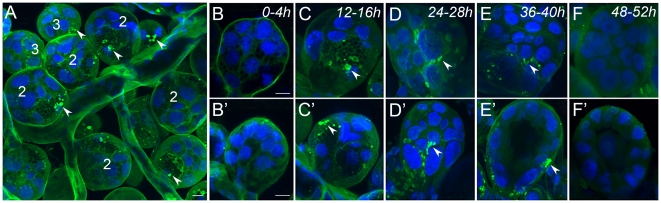
Dopamine immunoreactivities in the salivary glands of female ticks during various feeding phases. (A) A region of salivary glands with clustered acini II (labeled 2) and III (labeled 3) at 12–16 h after attachment. (B to F) Acini II at 0–52 h after attachment to the host and (B’ to F’) acini III at the same feeding 4ases. Note that positive staining (green marked with arrowheads) was detected in both acini II and III in the vesicles and their surrounding regions, but only at 12–40 h post-attachment. The blue color shows nuclei stained with DAPI. The images are composites of multiple confocal layers of thicknesses of 20 µm (A) or 2–5 µm (B to F’). Scale bars equal 10 µm.

In female *I. scapularis*, DA immunoreactivities were found in the large cells located in the basal region of acini II and III, respectively, but not in acini I (Figure 2A C–E’). One to two and three to four basal cells were immunoreactive in acini II and III, respectively. Within the basal cells, only subsets of large vesicles (∼2–5 µm in diameter) or their surrounding regions were strongly stained. Sampling at 12-hour intervals over the first 48 hours and daily samplings afterward until the repletion, which happened in day 7 or 8 after the onset of blood feeding showed that DA production in the basal cells occurred between 12 and 40 hours (Figure 2A–F’). DA immunoreactivities in other phases were not detected. The numbers of positive individual females were as follows: unfed (0/8, number positive/number tested individuals), 0–4 h (0/8), 12–16 h (3/8), 24–28 h (4/8), 36–40 h (6/13), 48–52 h (0/8), 3^rd^ day (2/9), 4^th^ day (0/8), 5^th^ day (0/8), 6^th^ day (0/9), and repletion stage (0/6).

### Expression pattern of the D1 receptor

**Figure 3:**
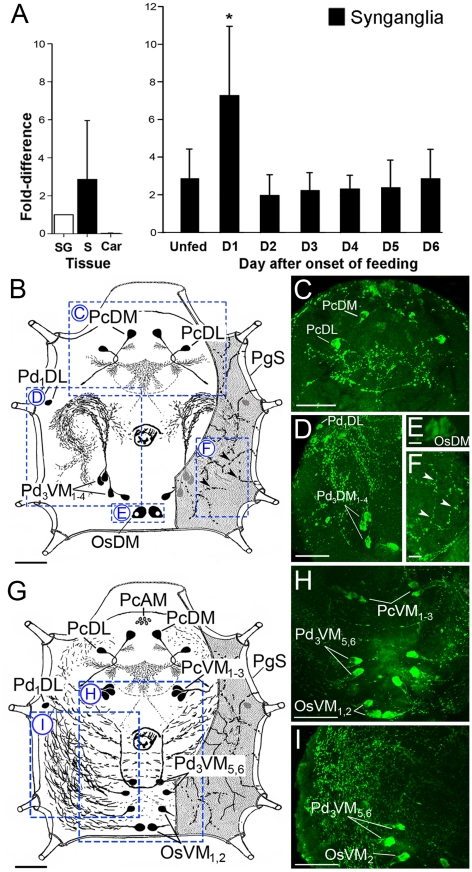
Expression patterns of the D1 receptor. (A) Quantitative RT-PCR showing the transcript levels of D1 in different tissues of females fed for 6 days (left panel) and temporal changes after the onset of feeding in synganglia (right panel). The averages and standard deviations of three biological replicates are shown. Asterisks (*) are for p<0.05 in ANOVA-Tukey’s post hoc means comparison test. More details are discussed in Materials and Methods. S, synganglia; SG, salivary glands; Car, carcass. Schematic diagrams showing D1 receptor neurons in the synganglia of unfed female (B) and after repletion (G). Blue boxes with dotted lines show immunohistochemistry in the right panel: (C) dorsal protocerebral region, (D) pedal region, (E) opistosomal neurons on the dorsal side, (F) periganglionic sheath surface of the dorsal-pedal region showing the axon terminals arborization, (H) vetral protocerebral, pedal and opistosomal region and (I) pedal and opistosomal region containing rich axonal arborization. Arrowheads in F indicate axonal arborization on the periganglionic sheath surface. The first two letters refer to the position of each D1 positive neuron in a specific lobe of the synganglion protocerebral (Pc), pedal 1–4 (Pd1–4), opisthosomal (Os), and the letters that follow refer to the anatomical location of the neuron: dorsal (D), ventral (V), anterior (A), medial (M) or lateral (L), and periganglionic sheath (PgS). The numbers in subscripts after the last letter refer to number of neuron pairs. Scale bars in B to D and G to I equal 50 µm and in E and F equal 10 µm.

**Figure 4:**
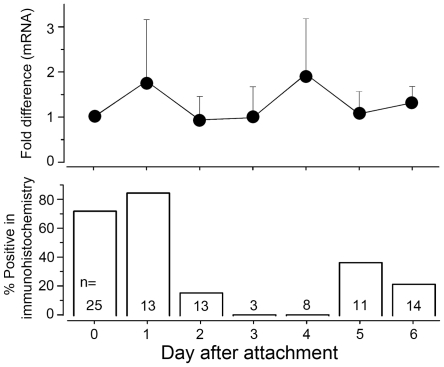
Figure 4. The levels of mRNA and protein of the D1 receptor in salivary glands. Quantitative RT-PCR showing the transcript levels of the D1 (upper panel) and the percent positive responses in the D1 immunohistochemistry (lower panel) are presented for females salivary glands fed for 6 days. The quantitative RT-PCR shows the averages and standard deviations of three biological replications.

Tissue-specific quantitative real time reverse transcriptase PCR (qRT-PCR) revealed that D1 receptor mRNA was abundant in the salivary gland and the synganglion of partially fed (6th day) females, while low levels of mRNA were also found in carcasses (Figure 3A, 4). Further examination of the SGs from different feeding phases showed that the D1 receptor mRNA was constitutively detected in the SG from pre-blood feeding to repletion of the tick. The mRNA level in the SG fluctuated between one to two-fold during the blood feeding. The D1 receptor transcript in the synganglia was significantly increased (7-fold) in the 1st day of feeding (Figure 3A, 4).

Nomenclature of neurons followed those used for the hard tick, *Rhipicephalus appendiculatus* and *I. scapularis* previously published [12], [13]. The first two letters refer to the position of each D1 positive neuron in a specific lobe of the synganglion protocerebral (Pc), pedal 1–4 (Pd1–4), opisthosomal (Os), and the letters that follow refer to the anatomical location of the neuron: dorsal (D), ventral (V), anterior (A), medial (M) or lateral (L).

D1-like immunoreactivity in the synganglion was found in a cluster of three pairs of small anterior neurons in the protocerebrum (PcAM). Two pairs of neuronal cells (PcDM, PcDL) were found in the dorsal protocerebrum and three pairs of PcVM_1,3_ on the ventral protocerebrum. One pair of small neurons (Pd_1_DL) was found in the pedal ganglia I (Figure 3B, C, G). We observed six pairs of cells (Pd_3_VM_1–4_ and Pd_3_VM_5–6_) in the ventral side of pedal ganglia 3 (Figure 3B,D, G-I). The Pd_3_VM_1–4_ cells had bilaterally symmetric projections directed anteriorly toward the pedal ganglia III and arborized in a loop in each pedal ganglion I–IV. Strong stains were also found in two large neuronal cells in the medial region of the opistosomal ganglia (OsDM) and two smaller pairs on the ventral side (OsVM_1,2_) (Figure 3B,E,G,H). Immunoreactive axons terminals exhibited arborizations in the surface of the periganglionic sheath (Figure 3B,F). Among the immunoreactive patterns of unfed female synganglion, PcDM, PcDL, Pd_1_DL and OsDM cells were always found, but Pd_3_VM_1–4_ cells were found in only three positive of six tested synganglia. On the other hand, synganglia of 6^th^-day fed and fully engorged females showed a reaction only in PcAM, PcVM_1–3_ Pd_3_VM_5,6_, OsDM (only in 6^th^-day fed females), and OsVM_1,2_ neurons. In 6^th^-day fed females, only a weak axonal network was observed inside the synganglia, while strong reactions in the cell bodies and their axonal projections in all regions within the synganglia were observed for the fully engorged females (Figure 3G,I).

**Figure 5:**
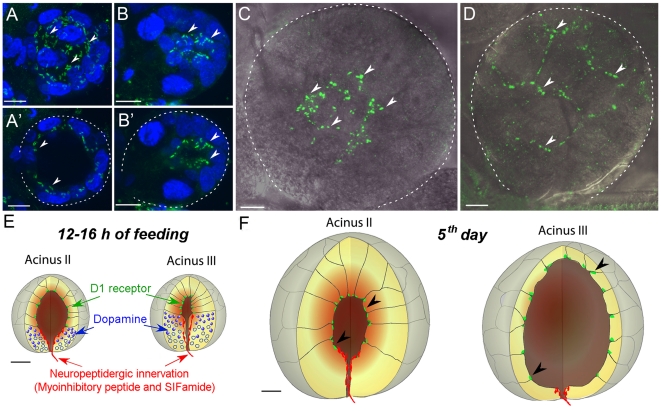
Immunohistochemistry for D1 receptor in the salivary glands. (A) Whole acinus II of unfed female shown as a 27 µm-thick composite image or (A’) a selected thin optical layer for a 10 µm thick demonstrating the apical location of the D1 receptor immunoreactivities. (B) Whole acinus III of an unfed female shown as a composite image of 34 µm thickness or (B’) a selected thin optical layer for a 5 µm thick demonstrating the apical location of the D1 receptor immunoreactivities. (C) Acini II and (D) III of a female at the 5th day post-attachment. Ten-micrometer-thick confocal composite images overlaid with the differential interference contrast images are shown. Schematic diagram showing the D1 (green), dopamine (blue), and neuropeptidergic innervation (red) [13] in acini II and acini III of the 12–16 hour fed female (E) and 5th day post-attachment in a female (F). Arrowheads indicate the scattered patches of D1 receptor reaction on the luminal side of the acini. Doted lines in A’, B’, C, and D indicate the boundary of an acinus. Blue in A to B’ is nuclei stained with DAPI. Scale bars are equally for 10 µm.

Strong positive signals in the SG were observed in the pre–blood feeding stage (Figure 4, 5A–B’, E,). A weaker signal was also found in the SG in the 1st and 2^nd^ day after the initiation of feeding in some individuals. The staining completely disappeared in the 3^rd^ and 4^th^ day, and staining reappeared in some individuals’ SGs on the 5th and 6^th^ day after attachment (Figure 4, 5C, D, F). Then, no positive signals were observed on the 6^th^ day or 7^th^–8^th^ days after repletion. The numbers of positive individuals were as follows: unfed (18/25, positive/tested individuals), 1^st^ day (11/13), 2^nd^ day (2/13), 3^rd^ day (0/3 tested), 4^th^ day (0/8), 5^th^ day (4/11), and 6^th^ day (3/14) (Figure 4).

D1 expression was limited only to acini II and III, but not found in acini I. Rod-shaped immunoreactive patches (∼2–3 µm long) on the luminal surface were clustered in the junctions between cells (Figure 5A–F). In the acini III, the patches of staining were limited to the apical region of the acinus and excluded the basal cells (Figure 5B,B’, D–F).

### D1 receptor reporter assays

**Figure 6:**
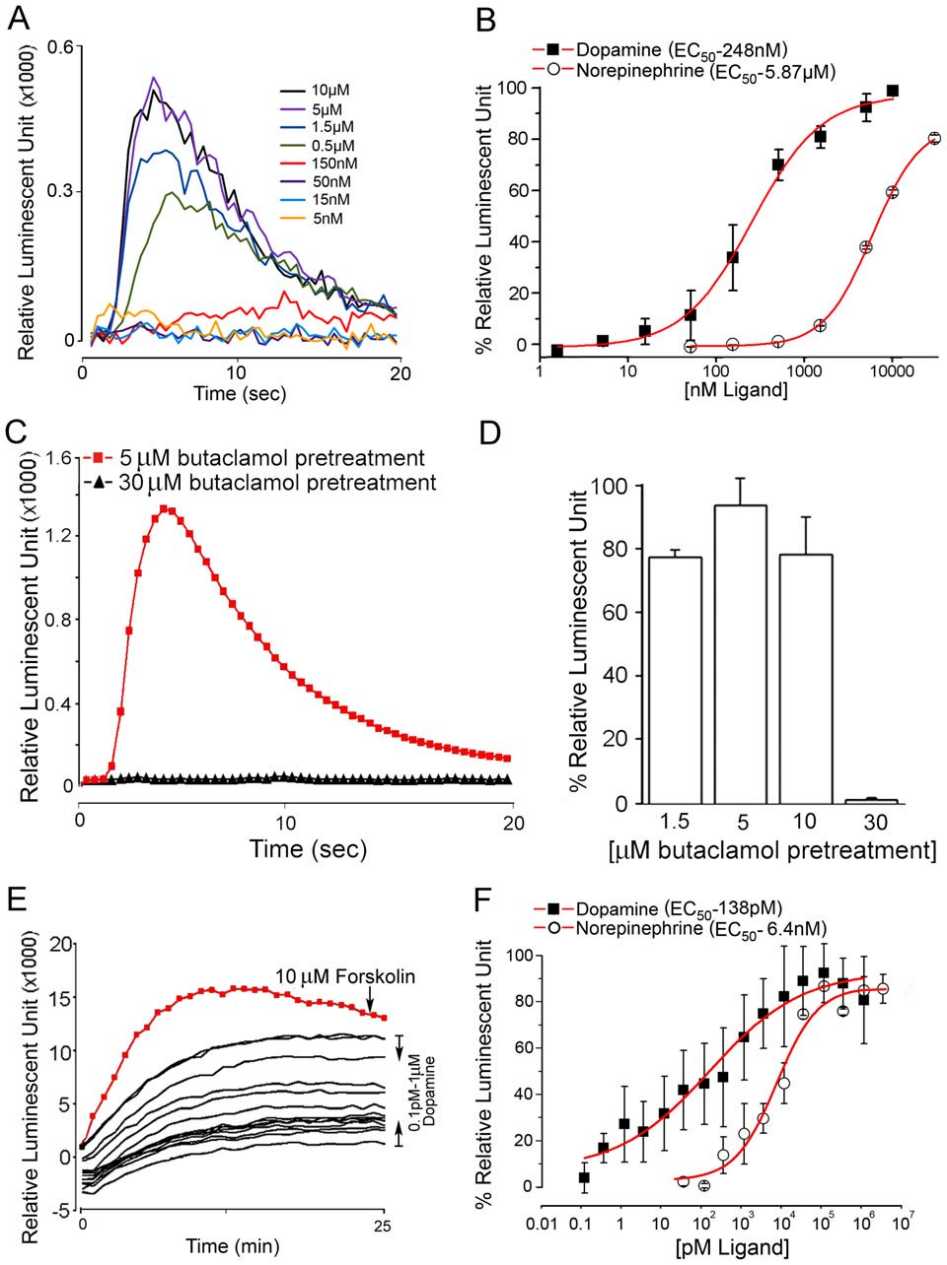
Reporter assays for D1 receptor showing DA-activated calcium mobilization and cAMP elevation. (A) Luminescent reporter calcium assay showing typical responses to varying doses (5 nM to 10 µM) of DA. (B) Dose–response curves of the CHO cells transfected with the D1 and aequorin constructs and treated with DA and norepinephrine. Antagonistic effect of butaclamol on the D1 receptor. (C) Luminescent responses to DA (3.3 µM) in the CHO cells expressing D1 receptor and aequorin. The cells were pretreated with 5 or 30 µM butaclamol (red and black lines, respectively) for 15 minutes. (D) Percent responses to DA (3.3 µM) when the cells were pretreated with different concentrations of butaclamol. Note that only 30 µM butaclamol was effective in blocking the DA action on the D1 receptor. (E) Luminescent reporter cAMP assay (GloSensor) showing typical responses to varying doses (0.1 pM to 1 µM) of DA. (B) Dose–response curves of the HEK cells transfected with the D1 and GloSensor constructs for DA and norepinephrine. Bars indicate standard errors for a minimum of four replicated plates.

A receptor assay, in which the reporter detects downstream calcium mobilization in chinese hamster ovary (CHO) cells upon activation of the D1 receptor, showed robust responses to various doses of DA and a low-level response to norepinephrine. More than 20-fold–higher activity of DA (EC_50_ = 248 nM) was found compared to that of norepinephrine (EC_50_ = 5.87 µM) (Figure 6A,B). Octopamine and pilocarpine did not produce any detectable response up to 10 µM. A 15-min preincubation with a high concentration (30 µM) of butaclamol, a dopamine receptor antagonist, completely abolished the DA-mediated response (at 3.3 µM) in the calcium reporter assay, while preincubation with 1.5 µM, 5 µM, or 10 µM butaclamol did not show significant effects on the response to treatment with dopamine (Figure 6C,D). Transfection with only the reporter aequorin construct did not result in any response to DA or norepinephrine.

Another reporter system monitoring the cAMP production in human embryonic kidney (HEK) cells indicated that the D1 receptor is also coupled to the pathway for cAMP production, a presumed adenyl cyclase–mediated pathway. DA showed the highest activity while norepinephrine was also active on the D1 receptor (EC_50_ = 138 pM and 6.4 nM, respectively) (Figure 6E,F). As seen in the calcium reporter assay, octopamine and pilocarpine did not show any activity up to the concentration of 10 µM. In addition to the strong activities of DA and norepinephrine on the receptor, serotonin (10 µM) induced low levels of cAMP elevation (<20% compared to the response to 10 µM forskolin, an activator of adenyl cyclase), and tyramine and metaergoid (10 µM) induced moderate responses (50–70% of the response to 10 µM forskolin, Figure S1). Transfection with only the reporter Glo-sensor showed a low response (cAMP elevation) to DA and norepinephrine at 10 µM (8% and 18% of the response to 10 µM forskolin, respectively), indicating the presence of endogenous receptor(s) in the HEK cells activating the reporter system, though the response was small enough that it did not mask the D1 receptor response to DA in this study.

## Discussion

The dopamine mediated tick salivary secretion has been widely used as an experimental tool to study tick salivary secretion and relatively well characterized for the physiology (reviewed in [7], [14]). This is the first molecular and functional characterization of a dopamine receptor in ticks. Our study reveals a novel paracrine DA system controlling the tick SG activity at the early blood feeding phase. Investigation of the spatial and temporal dynamics of DA production and its receptors in the tick SG provides insights into our understanding of the molecular mechanisms underlying the control of the SG.

### DA in the basal cells of salivary gland acini at the early feeding phase

The presence of DA in the *I. scapularis* SG, which we demonstrated by immunohistochemistry, has also been previously shown in South African bont tick *Amblyoma hebraeum* by chemical analyses of the SG using chromatography followed by electrochemical detection and using a radioenzymatic assay with catechol-O-methyl transferase [11], [16]. In the same study [16], immunohistochemical staining of tyrosine hydroxylase, the enzyme responsible for the biosynthesis of DA, revealed “spindle-shaped bodies” in the acini II and III. Based on our confocal microscopy, the spindle-shaped bodies are similar to what we observed in the early phase of DA immunoreactivity, particularly in the region surrounding the subset of large vesicles (Figure 2A, C’) which were considered as nuclei previously [16]. These observations suggest that DA production is compartmentalized into a subcellular domain in the cytoplasm near a subset of vesicles, which permits localized transport of the DA into the secretory vesicles.

Temporal patterns of DA immunoreactivity, indicating that the production of DA occurs only at the time between 12 and 40 h post-attachment, do not agree with an earlier study done in *A. hebraeum*. In that study, large quantities of DA were found in the SG of the partially fed female tick, which is likely after more than 2 days of feeding based on the weight of the ticks specified in the publication [11], [16]. A remaining question is whether the discrepancy is caused by the differences in the species or in the experimental approaches. Interestingly, a recent electron microscopy study [17] described low-electron-density granules in a-cells of acini II released for the first 2 days of feeding and large, electron-translucent granules in d-cells of acini III luminally released in the first day after attachment. The basal locations of the a- and d-cells in acini II and III with large granules (vesicles) of a size of 4–6 µm and ∼2 µm, respectively [17], and the temporal dynamics match the dopamine immunoreactive vesicles we describe in this study. Therefore, we conclude that DA synthesis and vesicle-mediated luminal secretion in the basal cells of acini II and III occur at the early feeding phase. Furthermore, inconsistent DA immunoreactivity, for which ∼50% individuals were positive in the 12 to 40 hr feeding, suggests that the DA production and secretion occurs in a short period of time and tightly controlled.

It is also noteworthy that the neuropeptidergic axonal projections terminate adjacent to the basal cells of acini II and III (Figure 5E, F), while there was no detectable dopamine immunoreactive neuronal projections to the SG in this study. We previously described two neuropeptides, SIFamide and myoinhibitory peptides, co-localized in a pair of large protocerebral cells (PcSG) and in the nervous projections reaching to the acini II and III of the salivary glands [12], [13]. The anatomy of the nervous projection of the PcSG containing those neuropeptides suggests a possible role in neuropeptidergic control of dopamine cells located in the basal part of acini II and III (Figure 5E,F).

### D1 receptors at the luminal surface of the SG

Dopamine acts through membrane-associated G protein–coupled receptors. At least three different genes encoding the DA receptors exist in the genome of *I. scapularis*: D1, D1-like, and D2 receptors. The tick D1 receptor characterized in this study showed the classical characteristics of D1-family receptors in its heterologous expression. Ligand-activated elevation of cAMP, presumably through the Gαs subunit and adenyl cyclase, occurred at a low concentration of DA. The lowest effective concentration was 30 fM. However, the concentration–response curve had a very shallow slope, which results in the curve spanning more than a five-log scale and implies that the reporter system was not a simple result of a one-on-one biochemical interaction between ligand and receptor. It is possible to have complex receptor conformations and coupling efficiencies to the reporter in the heterologous expression system, though it is clear that the tick D1 receptor responded to DA with a high sensitivity. In addition, the receptor also presumably acted through a presumed Gαq -coupled pathway, which induced intracellular calcium mobilization in the heterologous expression system. Relatively higher concentrations of DA were required for the calcium response compared to those required for elevation of cAMP.

Earlier studies in the SG response to DA have shown that the DA action occurs both through the activation of adenyl cyclase [10] and through calcium mobilization (both intra- and extra-cellular calcium) [18]. Subsequently, a model for DA-induced signal transduction in the tick SG was proposed [14], in which DA activates two independent signaling pathways: cAMP-dependent signal transduction leading to fluid secretions, and a calcium–dependent signaling pathway activating prostaglandin E2 production and eventually leading to secretions of other protein components in the salivary secretions. The DA action on the salivary secretion was blocked by a general DA receptor antagonists butaclamol [19]. The tick D1 receptor acting through both cAMP and calcium elevation and its inhibition by butaclamol described in this study are similar to the model proposed for the DA receptor in the tick SG, although a cautious interpretation of the results from the heterologous expression system is needed.

Previous studies of DA actions on the SG have focused on the middle feeding phases, while the SG in the phases before feeding or after the completion of feeding did not show the salivary secretion upon bath treatment of DA on the isolated SG [8]. The D1 receptor investigated in this study probably does not mediate the DA-activated salivary secretion in the middle feeding phases, based on phase-specific expression patterns shown by immunoreactivity. We propose that the D1 receptor which is expressed in the SG at the early feeding phase is the major receptor mediating the luminally secreted DA from the basally located cells of acini II and III.

Other possible functions of luminally released DA and the D1 receptor can be predicted based on our observation. We propose that the luminally released DA could be secreted into the host as a salivary component for vasodilation of the feeding site. DA may also have direct effects on pathogenic organisms. In *Borrelia burgdorferi*, an induction of the expression of outer surface protein A in response to the host catecholamines is known to facilitate the colonization in the tick midgut [20]. Furthermore, previous studies and our preliminary work indicating the presence of multiple dopamine receptors in the SG, suggest multiple functions of DA in the control of the SG through different types of receptors. We also found changes in the D1 receptor staining patterns in the synganglion, including its increased accumulation in the axonal projections that arborize in the synganglion at the end of the feeding phase (Figure 3G,I). These results imply that dopamine has dynamic roles in the neuronal system over the tick feeding phases. This study characterizing the source of DA and D1 receptor in the SG of tick strongly supports the paracrine function of DA in the SG and lays a foundation for understanding the functions of DA in tick physiology during host attachment.

Functional dissection of the DA signaling pathways in control of the SG through multiple receptors requires combinatory experimental approaches using pharmacology and RNA interference (RNAi). Unfortunately, our extensive efforts on the RNAi of the D1 receptor have not been successful in sufficient suppression of the transcript, which was confirmed by qRT-PCR and immunohistochemistry. Expanded work on other dopamine receptors and development of physiological assays on the SG will help to decipher the DA actions on the tick SG.

## Materials and Methods

### Experimental animals and chemicals

Unfed adult ticks, *I. scapularis*, were obtained from the tick-rearing facility at Oklahoma State University. A colony of *I. scapularis* was kept in polypropylene vials (9×2.5 cm) with the openings covered by cotton plugs. Each vial contained approximately 30 individuals of both females and males and a small piece of filter paper (4×1 cm). These vials were kept in a dark, humid chamber at 4°C. Ticks were fed on experimental New Zealand White rabbits (Myrtle’s Rabbitry, TN). Rabbits were cared for in accordance with the guidelines approved by the Institutional Animal Care and Use Protocol (IACUC approval no. 2721) of Kansas State University. Chemicals used in this study were: dopamine hydrochloride (Sigma), DL-octopamine hydrochloride (Fluka), pilocarpine nitrate salt (Sigma), (±) –norepinephrine (+)-bitartrate salt (Sigma), 98% forskolin (Fluka) from *Coleus forskohlii*, serotonin hydrochloride (Sigma), tyramine hydrochloride (Sigma), and methylergonovine maleate salt (Sigma).

### Gene cloning and sequence analysis

Gene predictions for the dopamine receptor were based on the results of blast searches in VectorBase (www.vectorbase.org). Initial search results for the matches of highly conserved regions were used for further analyses of the database and for designing primers to use in polymerase chain reaction (PCR). PCR products were cloned into the pGEM-T-easy vector (Promega, Madison, WI) and sequenced. The primers used for amplification of the D1 gene were as follows: forward, 5′-GAAAGTCGGATGTGTTGCTCCA-3′, and reverse, 5′-AGTACACTCGCTGCTATATGGC-3′. Transmembrane segments were predicted by using TMHMM Server v.2.0 (http://www.cbs.dtu.dk/services/TMHMM/). Phylogenetic analyses of putative *I. scapularis* dopamine receptors and related GPCRs were based on sequence alignments using CLUSTAL W2 [21]. Sequences were trimmed to contain conserved transmembrane segments one to seven. The tree was constructed with MEGA4 [22] using the neighbor-joining method with 500 bootstrap replicates.

### Quantitative real-time reverse transcriptase PCR (qRT-PCR)

RNA for qRT-PCR was extracted from dissected SGs and synganglia. The seven different samples represented tick feeding phases were unfed ticks and ticks collected daily for 6 days of attachment. The organs from eight individuals were pooled for each biological replication. All data presented are for three biological replications unless specified otherwise in the figure captions.

Total RNA was extracted by Trizol reagent (Invitrogen) and treated with Turbo DNase (Ambion) or RNeasy Plus Micro Kit (Qiagen) with incorporated on-column DNase treatment to obtain DNA-free RNA. Reverse transcription using Superscript III (Invitrogen) with polyT primer was followed by real-time PCR using SYBR premix Ex Taq (Takara Bio). Primers targeting the 278-bp amplicon were DopR1-F: TGCCTGGCCATCTACACGGATC and DopR1-R: TTGGTCATCCAGCGGCCGTAGC. The *I. scapularis* ribosomal protein S4 (GenBank Accession No. DQ066214), which was determined to be the most suitable for the stabilities among different tissues and feeding stages in our preliminary study (data not shown), was used as the reference gene. The 174-bp product was amplified with the following primers: RPS4-F: AGGCCAAGTACAAGCTGTGC and RPS4-R: CGAACTTGATGTAGTCGTCG. mRNA level was quantified using the ΔΔCt method, corrected by the amplification efficiency of each target gene, and expressed as a fold difference [23]. Data were analyzed by one-way analysis of variance and Tukey’s post hoc means comparison in Origin v.7 (OriginLab).

### Immunohistochemistry

Ticks were dissected in ice-cold phosphate-buffered saline (PBS: 137 mM NaCl, 1.45 mM NaH_2_PO_4_, 20.5 mM Na_2_HPO_4_, pH 7.2). The synganglia and the salivary glands were cleaned of remaining blood and fixed in Bouin’s solution (37% formaldehyde and saturated solution of picric acid 1:3) or a mixture of 4% paraformaldehyde and 2% glutaraldehyde at room temperature for 2 hours or 4°C overnight, respectively. The fixed samples were washed in PBS containing 1% Triton X-100 (PBST). The tissues were then preadsorbed with 5% normal goat serum (Sigma) in PBST for 10 minutes and subsequently incubated with anti-dopamine (ab888, Abcam) and anti-D1 (see below) antibodies (1:300 each) for 2 days. The preadsorption step was omitted for dopamine immunohistochemistry. After three washes with PBST, the tissues were incubated overnight in goat anti–chicken or anti–rabbit IgG antibody conjugated with Alexa Fluor 488 (Molecular Probes). The tissues were washed in PBST and mounted in glycerol containing 300 nM 4′,6′-diamino-2-phenylindole (2 µg ml^−1^; Sigma). Images were captured on a confocal microscope (Zeiss 510 Meta). Schematic drawings were made in Adobe Photoshop 7.0 or Canvas 8.0. Data presented are only for multiply repeated staining patterns, and they are specified in the figure captions.

Antibody against the D1 dopamine receptor was raised in a chicken (Genescript, New Jersey). An antigenic peptide was designed for the region predicted to have high surface probability and antigenicity and low probability of post-translational modification. The carboxy-terminal 20 amino acid residues, CEVDTRVEISVKSVGEISAI, were chemically synthesized and conjugated to keyhole limpet hemocyanin. The final bleed was used for affinity purification. Nomenclature of neurons followed that used for the hard tick *R. appendiculatus* and for *I. scapularis*, as previously published [12], [13]. The first two letters refer to the position of each D1-positive neuron in a specific lobe of the synganglion: protocerebral (Pc), pedal 1–4 (Pd1–4), or opistosomal (Os); and the letters that follow refer to the anatomical location of the neuron: dorsal (D), ventral (V), anterior (A), medial (M), or lateral (L). The immunohistochemistry with pre-immune serum for anti-D1 receptor showed no specific staining pattern in the pre-feeding stage, demonstrating the antibody specificity (Figure S2). The blast search of the *I. scapularis* genome using the antigenic peptide sequence as query did not find any significant matches except to the D1 receptor gene.

### Receptor reporter assays

The full-length open reading frame (ORF) of the D1 receptor was inserted into the plasmid pcDNA3.1(+) (Invitrogen). For calcium mobilization assays, transient expression of the D1 receptor was assessed using the reporter aequorin (cytoplasmic aequorin, [24]) in CHO cells. Luminescence assays were performed in opaque 96-well microplates (Corning) using an Orion microplate luminometer (Berthold Detection Systems). Various doses of ligands in 50 µL were plated in each well. The changes in luminescence were monitored for 20 s immediately after injection of cells into the well (∼15,000 cells in 50 µL). The luminescent response was integrated over time and normalized to the largest positive control response in each plate (10 µm DA for the D1 receptor) after background subtractions. The effect of the antagonist butaclamol was measured after a pre-incubation of the cells for 15 min at room temperature with different concentrations of butaclamol. Subsequently, the cells were treated with 3.3 µM dopamine, the dose inducing the maximum response, and the 20-s luminescent response was measured.

For cAMP response assays, we used the non-lytic GloSensor cyclic AMP assay kit (Promega) for monitoring intracellular cAMP in live cells. Co-transfections of the plasmid containing D1 receptor and the plasmid with GloSensor were performed in HEK cells, Invitrogen). Cell suspensions were pre-equilibrated with 1% GloSensor substrate (Promega) before the test. The same luminometer described above was used to detect the luciferase activity. Ligands being tested were plated in each well of the 96-well plate. Following the injection of cells (∼10^5^ in 50 µL) into a well, the changes in luminescence were monitored in ∼1-min intervals for 25 min. Relative luminescence at 15 min after the incubation with various doses of ligands was normalized by the largest positive control response in each plate (300 nM DA for the D1 receptor) after background subtractions. Each plate contained at least two replica wells for a dose of a ligand in all plate assays.

Cells were grown in Ham’s DMEM-F12 medium supplemented with 10% FBS, 100 U/ml penicillin, 100 mg/ml streptomycin, and 2.5 mg/ml fungizone. Transfections of reporter and receptor constructs were performed using FuGene HD (Roche) according to the manufacturer’s protocol at a ratio of DNA to FuGene of 3:2. The responses mediated by endogenous receptors from the CHO and HEK cell lines were determined by mock transfection with only the reporter construct. All ligands were dissolved in distilled water except forskolin in dimethyl sulphoxide (DMSO) (Sigma) for the stock solutions. Further dilutions were made in the working solution: Ham’s DMEM-F12 media supplemented with 0.1% bovine serum albumin (BSA) (Millipore) for calcium assay or CO_2_-independent media (Invitrogen) for cAMP assay.

## Supporting Information

Figure S1 **Agonistic activities of different compounds on the D1 receptor in the reporter assay measuring the induced elevation of cAMP.** Data are percent luminescent values normalized by the response to 10 µM forskolin.
(PDF)

Figure S2 **Pre-immune negative controls for D1 receptor.** (A) acinus II and (B) acinus III of unfed female. Scale bar 10 µm. Doted lines indicate the boundary of an acinus.
(PDF)
