# Supplementary material for: Evidence for D1 Dopamine Receptor Activation by a Paracrine Signal of Dopamine in Tick Salivary Glands
Source: PLoS One. 2011 Jan 31;6(1):e16158. doi: 10.1371/journal.pone.0016158 (PMC3031531; doi:10.1371/journal.pone.0016158)

Figure S1. Agonistic activities of different compounds on the D1 receptor in the reporter assay measuring the induced elevation of cAMP. Data are percent luminescent values normalized by the response to 10 μM forskolin.

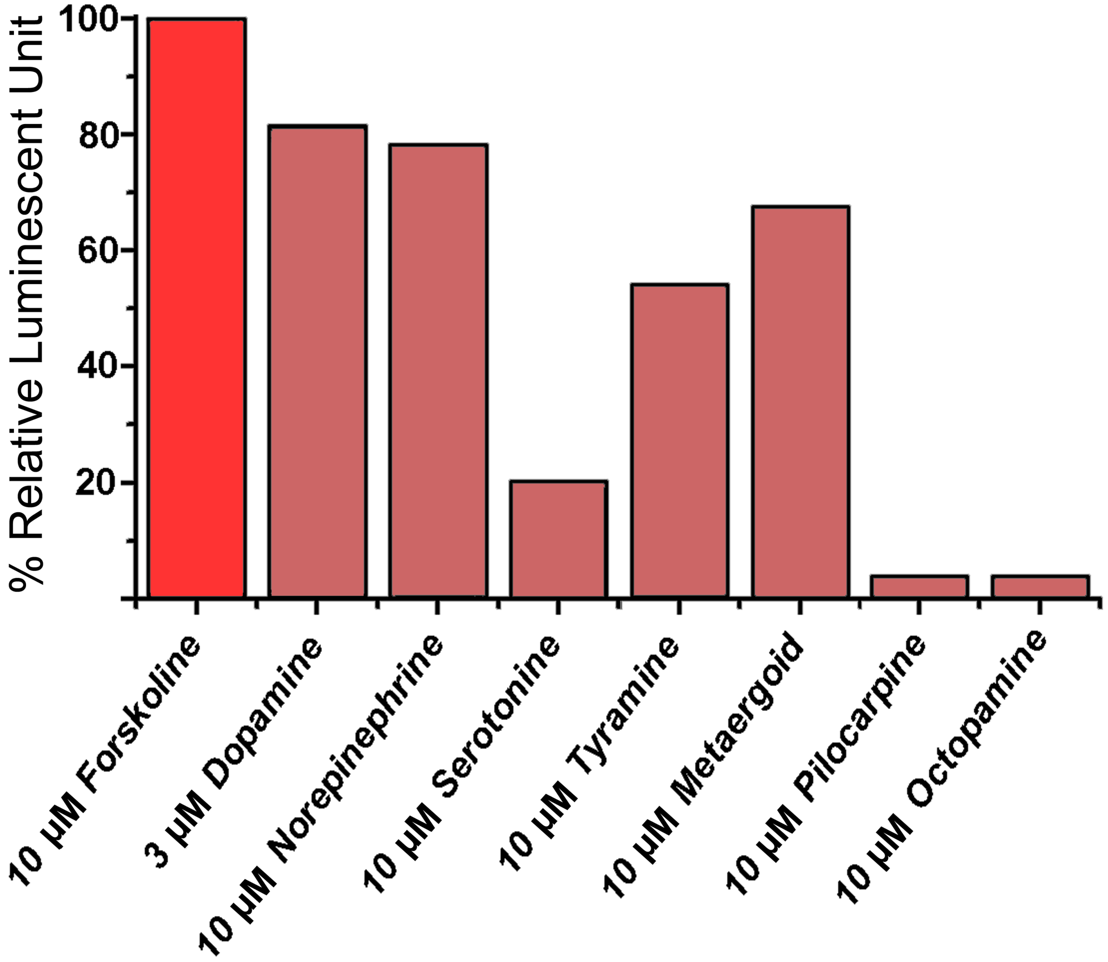

Supplement: Figure S1 — Agonistic activities of different compounds on the D1 receptor in the reporter assay measuring the induced elevation of cAMP. Data are percent luminescent values normalized by the response to 10 µM forskolin. (PDF) [file pone.0016158.s001.pdf]
