# Supplementary material for: Evidence for D1 Dopamine Receptor Activation by a Paracrine Signal of Dopamine in Tick Salivary Glands
Source: PLoS One. 2011 Jan 31;6(1):e16158. doi: 10.1371/journal.pone.0016158 (PMC3031531; doi:10.1371/journal.pone.0016158)

Figure S2. Pre-immune negative controls for D1 receptor. (A) acinus II and (B) acinus III of unfed female. Scale bar 10 μm. Doted lines indicate the boundary of an acinus.

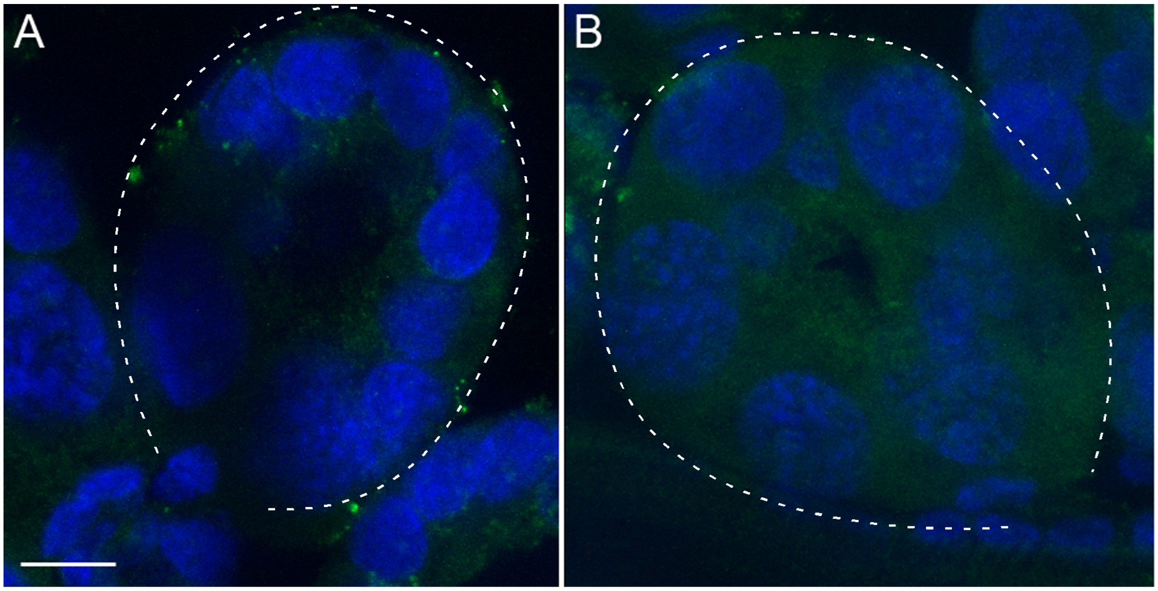

Supplement: Figure S2 — Pre-immune negative controls for D1 receptor. (A) acinus II and (B) acinus III of unfed female. Scale bar 10 µm. Doted lines indicate the boundary of an acinus. (PDF) [file pone.0016158.s002.pdf]
